# Key Aggregation Cryptosystem and Double Encryption Method for Cloud-Based Intelligent Machine Learning Techniques-Based Health Monitoring Systems

**DOI:** 10.1155/2022/3767912

**Published:** 2022-04-21

**Authors:** Khalid K. Almuzaini, Amit Kumar Sinhal, Raju Ranjan, Vikas Goel, Rajeev Shrivastava, Awal Halifa

**Affiliations:** ^1^National Center for Cybersecurity Technologies (C4C), King Abdulaziz City for Science and Technology (KACST), Riyadh 11442, Saudi Arabia; ^2^Department of Computer Science and Engineering, Institute of Engineering and Technology, JK Lakshmipat University, Near Mahindra SEZ, Ajmer Road, Jaipur 302026, India; ^3^School of Computing Science and Engineering, Galgotias University, Greater Noida, India; ^4^Department of IT, KIET Group of Institutions, Delhi-NCR, Meerut Road, Ghaziabad-201206, India; ^5^Department of ECE, Princeton Institute of Engineering and Technology for Women, Hyderabad 500088, Telangana, India; ^6^Kwame Nkrumah University of Science and Technology, Kumasi, Ghana

## Abstract

Cloud technology is a business strategy that aims to provide the necessary material to customers depending on their needs. Individuals and cloud businesses alike have embraced the cloud storage service, which has become the most widely used service. The industries outsource their data to cloud storage space to relieve themselves of the load of dealing with redundant data contents. This must be protected to prevent the theft of personal belongings, and privacy must be improved as well. Different research projects have been suggested to ensure the safe management of the information included within the data content. The security of current research projects, on the contrary, still needs improvement. As a result, this method has been suggested to address the security concerns associated with cloud computing. The primary goal of this study effort is to offer a safe environment for cloud users while also increasing the profit of cloud resource providers by managing and securely delivering data contents to the cloud users. The bulk of sectors, including business, finance, military, and healthcare industry, do not store data in cloud-based storage systems. This technique is used to attract these kinds of customers. Increasing public acceptance, medical researchers are drawn to cloud computing because it allows them to store their study material in a centralized location and distribute and access it in a more flexible manner. They were collected from numerous individuals who were being evaluated for medical care at the time. Scalable and enhanced key aggregate cryptosystem is a protected data protection method that provides highly effective security in the healthcare industry. When parties interested in a dispute disagree on the outflow of sensitive information, this technique manages the disputes and ensures the data security deployment of a cloud-based intelligent health monitoring system for the parties involved. The encrypted data structure of medical and healthcare prescriptions is recorded as they move through the hands of patients and healthcare facilities, according to the technique recommended. The double encryption approach is used in order to raise the overall degree of security. An encryption class is created by referring to the Ciphertext ID during the encryption procedure. The keyholder is a master secret key that facilitates in the recovery of the secret keys of various monsters and creatures by acting as a conduit between them. It is transferred and stored as a single aggregate for the benefit of the patient or customer in order to make decryption more convenient and efficient. A safe connection between cloud-based intelligent health monitoring systems and healthcare organizations and their patients may be established via the use of a key aggregation cryptosystem and a double encryption approach, according to the researchers. Because of this, when compared to earlier techniques, the findings reveal that the research methodology provides high levels of security in terms of confidentiality and integrity, in addition to excellent scalability.

## 1. Introduction

As with any relationship of agreement between two parties, cloud security is similar in that it requires confidence in the relationship of agreeing with each other, which manifests itself as sensitive data both statically and during transmission. Customers and service providers that interact with a cloud computing environment need a high level of confidence in the environment [1]. Because cloud computing incorporates a variety of local methods and draws in a large number of people with a variety of backgrounds, it has the potential to become very complicated. Private information on a differential scale is presently receiving a great deal of attention as a potential alternative for partition-based privacy models for privacy preservation. When it comes to cloud computing, security may be achieved via a variety of methods such as authentication, data integrity, and confidentiality [2]. This consists of providing individuals with on-demand and prior reservation-based access to combine computational and other resources, autonomics, the ability to categorize resources from potentially disparate clouds to produce the results of workflows, and the appropriate level of security and privacy. A powerful cloud-based operating system provider, a productivity suite provider, postal service providers, and storage providers are all investigated using this method [3]. A cataract is a significant and rapidly increasing worldwide health problem. The goal is to prevent cataracts from progressing further and to discover a treatment for them. The loss of privacy concerning the data of the users has an impact on the ability to provide the service properly in the cloud environment. Performing a comparative study of cloud-based privacy preservation methods provides a clear picture of the privacy issues and strategies that are being utilized for data preservation in the cloud. As a result, the data mining method coupled with optimization approaches is presented to address the issue of privacy preservation [4]. Consequently, the present study endeavours to resolve the security issues that have been previously addressed by introducing new methods. When it comes to avoiding security problems, the earlier-used methods provide superior results, but they may impair the overall performance in a variety of ways. This strategy is designed to maximize the security level and safeguard the cloud data contents in the study methods that have been recommended. The primary aim is to guarantee and offer a secure environment for data related to healthcare services and products. The fact that the apps are located in several places further adds to the public's worry about privacy [5]. In addition to storing data and providing flexible processing capacity, cloud computing helps to propel information technology (IT) to greater heights while requiring less initial capital expenditure. A company that operates its application in the public domain and outside of the firewall will raise knowledge about security and the issues that may arise as a result of that awareness. In the case of cloud computing, consumers may access resources from anywhere in the world at any time by connecting to the Internet, eliminating the need to deal with the real resource issues, such as physical and technical administration. Unlike traditional computer resources, cloud computing resources are dynamic and adaptable [6].

Because random number creation in symmetric key encryption techniques is readily recognized by the user, the One-Time Pad (OTP) generator is utilized by the majority of approaches in current research [7]. In cryptography, the One-Time Password (OTP) is an encryption method that cannot be broken. An encrypted plaintext is used in conjunction with a randomly generated secret key, also known as a one-time pad, in this kind of encryption [8]. In the next step, each bit or character of the plaintext is encrypted by adding it to the matching bit or character using a technique known as modular addition. Providing that the key is random, is at least as long as the plaintext, is never reused in whole or in part, and is kept secret, the resultant ciphertext will be almost difficult to decode and break into. It has also been shown that any cipher with the perfect secrecy property must employ keys that meet the same criteria as OTP keys to function properly [9]. One-time pads are information-theoretically secure in the sense that the encrypted message, referred to as the ciphertext, gives no information about the original message to a cryptanalyst other than the maximum length of the message that may be sent. A feature of the one-time pad that he coined “perfect secrecy” is that the ciphertext C contains no new information about the plaintext other than what is already known. Specifically, it is because every plaintext of equal length may be converted into any other plaintext if a genuinely random key is used only once, and all of the conversions are equally probable [9]. As a result, the chance of receiving a plaintext message *M* is the same as the likelihood of receiving a plaintext message *M* after the fact.

With the Diffie–Hellman key exchange protocol, it is possible to arrive at a shared secret key by exchanging text messages over an insecure medium without having to meet in person first. The Diffie–Hellman key exchange protocol is confined to the exchange of just one key between participants. In the absence of entity authentication, this protocol is susceptible to man-in-the-middle attacks and impersonation attacks, among other things. It has been noticed that Nanli's protocol, despite its efforts to remove the man-in-the-middle attack, is still vulnerable to the impersonation attack. This study proposes an enhanced key exchange strategy based on a third-party authentication system in order to deal with this vulnerability.

The entropy of the plaintext is denoted by *M*, while H(M|C) is the conditional entropy of the plaintext when denoted by the ciphertext. Perfect secrecy is a strong concept of cryptanalytic difficulty in the cryptographic community. Symmetric encryption methods that are now in use complicated patterns of substitution and transpositions [10]. It is not known if there may be a cryptanalytic method that can reverse these changes without knowing the key that was used during the encryption process, for the best of this presently in use, which is the best of the best. Asymmetric encryption methods are based on mathematical problems that are believed to be difficult to answer, such as integer factorization and discrete logarithms, which are both difficult to solve. The fact that these issues are difficult is not proven, and a mathematical breakthrough may render current systems susceptible to assault. OTP, in contrast to traditional symmetric encryption, is impervious to brute force assaults because it maintains complete secrecy, unlike ordinary symmetric encryption [11]. Attempting all keys just results in a slew of plaintexts, each of which is equally likely to be the real plaintext. When there is known plaintext, such as a portion of the message being known, brute force assaults are ineffective because the attacker is unable to get any information about the portions of the key that are required to decode the remainder of the message from the plaintext. All that will be revealed by these parts are just those parts of the key that correspond to the users, and since each user corresponds to a single part of the key, no portion of the key is reliant on any other part of the key [12]. Providing a safe environment for cloud users, as well as increasing the profit of cloud resource providers, is the primary goal of this study effort. This is accomplished via the secure handling and provisioning of data contents. Increasing public acceptance, medical researchers are drawn to cloud computing because it allows them to keep their study material in a centralized location and share and access it from anywhere at any time [4].

This technique, known as the Diffie–Hellman key agreement protocol, allows two parties to exchange secret keys over an unprotected channel without having to physically meet in person. The secret key is used for further encryption and decryption of the message and the cypher text, respectively, once they have been encrypted. When it comes to key management, there are several obstacles to overcome [13]. To ensure safe communication, every pair of users' need have a unique key, which is the first obstacle.

As a result, if a communication network has *n* users, then each member of the network must hold (*n* − 1) keys in their possession. A big number of members in a network make it very difficult to keep track of such a large number of keys in a secure manner. During the course of public key cryptography, the participant is required to identify two separate keys for the purposes of encryption and decryption [14]. These two keys should be multiplicative inverses of each other, and they should be the same. The most significant drawback of public key cryptography is that the encryption and decryption processes are both excessively slow. Man-in-the-middle attacks, in which a trusted third party takes on the role of the man in the middle, are also a concern in public key cryptography. In a man-in-the-middle attack, a third party (C) impersonates itself from A to B and B to A communication channels [14]. In this method, both parties communicate with one another via the intermediary of third-party C. The solution to this challenge is to provide a means of authentication between parties who are talking with one another.

On the subject of group key management, a number of systems have been proposed. Researchers examined the most current developments in security parameters in key management, as well as techniques for distributing session keys in a safe manner, and they also explored the topic of key refreshing. In addition to offering an efficient method for many-to-many and one-to-many communications, multicast or group communication facilitates the delivery of material on a broad scale.

In symmetric key cryptography, one of the most difficult problems to solve is the establishment of a secret key between two participants [15].

It is necessary to gather medical information from a variety of patients so that it may be evaluated to anticipate the information about a medical cure. It is critical to store and safely retrieve data so that a security breach does not result in an incorrect prediction or analysis. Health care data are stored and managed securely and confidentially in cloud computing storage, which is a concentrated area of storage [16]. Novel methods for protecting healthcare data stored in the cloud are being suggested in this study. By adopting new techniques, the potential security breach that might occur when sharing the cloud-stored healthcare data with various researchers is mitigated to the greatest extent possible. Because of the high amount of information being handled, it is not possible to ensure complete security improvement. Because of advancements in technology, the hacking process has become more accessible, which must be avoided to avoid data corruption and theft [17]. The current study studies do not address the privacy concerns that would arise if sensitive information, such as the legitimacy and ownership of the associated data, was disclosed.

It is necessary to exchange keys in a certain mathematical configuration in which there is no need for user authentication. Cas Cremers gave a presentation on how to enhance the standard for key management, which was based on his study.

Man-in-the middle attacks on the Diffie–Hellman protocol are common, and it is required to create a solution to remove man-in-the-middle attacks in order to ensure the safe transfer of the secret key between two parties using this protocol.

Several strategies have been proposed to provide the key exchange with user authentication in order to remove the man-in-the-middle threat by using hashing algorithms [18]. It is addressed in this work how the technique given by Nanli [19] for removing the man-in-the-middle attack may be used, the issue of impersonation by an authenticated member is discovered, and a solution to the identified assault is proposed.

The goal of a Diffie–Hellman key exchange is to provide parties with the option to generate a symmetric session key via an insecure communication channel. In addition, a symmetric key is exchanged with a session key for the purpose of encrypting and decrypting data. The discrete logarithm issue has an impact on the strength of the secret key produced in the Diffie–Hellman protocol [20]. In reference, the discrete logarithm issue is described as the amount of security that protects the deducing of the key from being compromised. Suppose “a” is a number, and the power modulo *p* of this number creates all integers from 1 to *p* − 1; in this case, “a” is known as a primitive root or a generator of the prime number “p.” The numbers generated from 1 to p − 1 are as follows: a (mod)p, a2 (mod)p,… ap-1 (mod)p.

These numbers, numbered from 1 to *p* − 1, create a permutation of sorts. For an integer *b*, b: bp, a prime number „p, and a generator „a of prime number „p, the expression b = ai (mod)p is produced as b = ai (mod)p, where 0 I *p* and 1 I *p* are positive integers (*p* − 1).

The issue of discrete logarithm of integer “b” on base “a” modulo *p* is referred to as I in this context. This value I may be represented by the notation d.loga, p (b).

Similar to the description in reference, the key exchange protocol is defined by utilising two public parameters, which are known as a prime number supplied as „q” and an integer given as “.” The integer “q” is generated by the provided integer “q.”

User A chooses a one-time random number (private key) XA such that XA q computes the public key parameter.

YA = [(X A)]mod q by multiplying YA by q. In a similar manner, user B chooses a new random integer as its private key, and user B computes its public key as YB = [(X B)]mod q by multiplying the private key by the modulus q.

X values are kept private on both sides, while the Y values are made publicly visible to the other user on the opposite side. The key for user B is determined as k = [(YA) (X B)]mod q, where q is the number of users.

## 2. Related Work

Hsiao Ying Lin and Wen GueyTzeng are two Taiwanese artists [21]. It was initially introduced as a technique known as distributed secured data storage, which counts the recovery process in a cloud database by the use of technologies known as encryption, according to its creators. It is possible to combine forwarding and encryption techniques, as well as an encryption mechanism, into a single package using this technology. There are a plethora of methods accessible, each of which includes retrieval approaches as well as cloud data storage capabilities. Tseng et al. [22], when it comes to discussing how to share data with other users in a safe, efficient, and adaptable manner in cloud data storage, have come up with a novel approach. It was discovered that the investigators employed a public key cryptosystem to produce ciphertexts that were all the same size input, which allowed them to grant operational decryption privilege to ciphertexts in response to a user's request for decryption rights. It has been shown that employing cryptography aggregate key techniques in cryptosystems, it is possible to compress the secret keys used in the system. As a result of utilising this strategy, which aids in the safe storage of data, the amount of storage space available will be reduced only when all users are using the same set of privileges and key sharing [23]. Because of the restrictions imposed by this method, it can only be used with certain kinds of encrypted communications. We calculated an effective pairing-based PPDP approach, according to Huaqun Wang and Zhang [24] in order to assure security in cloud data communications. When a customer is unable to do a remote check on their data in public clouds, they must be aware of their data ownership in public clouds. This technique is divided into three parts: the model of the PPDP system, the design process, and the security model. The notion of bilinear pairing is used to build and execute an efficient PPDP protocol, which is then put into practise. Performance and security studies have been conducted to ensure that this protocol is both efficient and secure [25].

In [26], the development of an attribute-based data interchange system for the smart grid is now underway. Not only has the data been evened out but also the access criteria have also been concealed from the view of grid staff throughout the course of the data exchange. Although both policy privacy and data privacy are protected under this method, the latter is more difficult to achieve. It is feasible to convey the access policy via the use of a number of arbitrary access equations. This increases the expressiveness of the policies as a consequence of the transformation. Additionally, the security is reinforced such that data that are to be transferred cannot be decrypted by a key generation centre that is not authorized to do so or by grid management systems that do not retain the data. Delegating the exceedingly labor-intensive procedures of decryption to more powerful grid-managed systems that are more efficient reduces the irregularities in the receivers' calculations as well as their overall performance. Gopinath and Geetha [27] are two of the most recent publications in this field. The development of an auditing framework for cloud storage systems, as well as an auditing protocol that is both effective and privacy-preserving, have both been presented in this study [28]. It has been improved to incorporate an auditing protocol to ensure that data dynamic operations are maintained in a way that is both provably safe and efficient in the arbitrary oracle modelling context. A trusted organizer or any other middleman is not required for the usage of this auditing protocol, which enables for batch auditing of several clouds and different owners [29]. The simulation findings and analyses reveal that the suggested auditing protocols are efficient and secure and that they, in particular, lower the computational expenses incurred by auditors in the course of performing their duties. In [19], a role-based encryption (RBE) paradigm, which integrated cryptographic approaches with a system known as role-based access control (RBAC), to protect sensitive information (RBAC) was suggested. On the basis of this approach, a secure RBE is provided which is based on hybrid cloud storage planning and provides an organization with the ability to store data safely in the public cloud while simultaneously maintaining the confidentiality of the company's information in a private cloud environment [30].

Waddington and Smith [31] addressed the difficulties and possible solutions associated with giving access to research results and storing them for long periods of time, which are mostly reliant on research data. Cloud storage and compute services are an ideal alternative for storing and preserving research data since they provide ready-to-use storage and computation services. Cloud infrastructure is more scalable and may be implemented on demand as compared to the construction of infrastructure inside a company, which frequently requires considerable upfront investment and extensive lead times [32]. The use of commercial cloud services, in particular, presents concerns about cost-effectiveness, trust, and governance, as well as obstacles with regard to service quality. In the case of data that are kept in the cloud, this approach identifies a collection of thorough case readings for scientists and researchers who are conducting a data-intensive study and demonstrates the questions that will be examined throughout the process. In the next step, the researchers evaluate the feasibility of developing a database system that meets all of the criteria. It is planned to implement a hybrid cloud, which would mix internally managed storage, cloud storage, and cloud services into a single unified storage area network (SAN) to improve performance. The content is sent to storage providers in accordance with a rule-based distribution technique. According to Lees et al. [33], a cloud-based storage system is presented that enables the outsourcing of dynamic data. The owner is able to preserve and retrieve data stored by the cloud service provider, as well as produce new data in the cloud storage system as required. Individuals may be certain that they are getting the most up-to-date version of the outsourced information thanks to their system, which is accessible only to authorized users. Kumar et al. [34] presented a CPABE system with multiple authorities that contains the following features: the system does not require a fully trusted central authority, and each attribute authority independently issues secret keys to users; second, each attribute authority can dynamically remove any user from its domain, ensuring that the revoked users are prevented from accessing subsequently outsourced data; and third, cloud servers can update encrypted data from the current period to the next period, ensuring that the revoked users are prevented from accessing subsequently outsourced data [35]. The integration of information and communication systems has helped a number of real-time and mission-critical applications, such as weather monitoring, flood detection, and emergency response, among others. Healthcare computer systems have gained a great deal of attention in recent years, and a growing number of institutions are implementing healthcare solutions to extend and enhance their services while keeping the total cost of ownership as low as possible [36].

Ultimately, the purpose of healthcare computer systems is to provide its users with easy and quick access to healthcare data and services, regardless of when or where they are accessed. This aim may be achieved in part via the use of cloud computing and its business model, which allows these services to be made accessible on a larger scale and at a more affordable price than would otherwise be feasible. The effect of this is that the majority of healthcare companies are apprehensive of using cloud-based solutions due to the possibility of data security and privacy concerns. Consequently, healthcare systems and services are now delivered through the public or private cloud in a cloud computing environment, rendering them vulnerable to a broad variety of security issues, including identity theft and fraud. A considerable chance exists that unauthorised users may seek to get access to sensitive health information, which might result in serious data security and privacy concerns. When it comes to healthcare data in cloud computing environments, the proposed concept describes a study of the security and privacy challenges that must be addressed. It also proposes a solution that will prevent unauthorised access to data by introducing an access control mechanism that can act as a security layer on top of all cloud service models, which can be implemented as a cloud service model. As a result, new techniques of securing healthcare data that are stored in the cloud are being examined. If new security approaches are used, they may help to avoid security breaches from happening when cloud-based stored healthcare data are shared with a large number of researchers. In light of the large quantity of information that must be managed, it is impossible to ensure comprehensive security enhancement. As technology advances, it will become simpler for hackers to infiltrate systems, which must be prevented in order to prevent data damage and theft from happening. The present research efforts are hindered by privacy issues, since sensitive information such as the validity and ownership of the linked data would be made public if the research work was released in its current state. Data owners have divided their current work files into a number of blocks, which will be retained in the index tree structure for future reference. As a consequence of the way the current technique is working, the following issues have arisen. Overhead in calculation has increased. Storage capacity is being used more often. Depending on whether or not the CSP is acting as a malicious provider, it is conceivable that the data kept on the server may get compromised. This must be avoided in order to ensure server security. The recovery of content from a cloud server that has gone down is a difficult task that must be conducted with more care in order to avoid content loss.

## 3. Materials and Methods

Cloud customers, as well as cloud service providers, have identified several difficulties [37]. Assurance of storage process and sharing are done in a secured manner [38]. Many current research projects have traditionally been made accessible in a secure cloud computing environment. However, as a result of maintaining a mostly conventional methodology, it fails to provide security. The previous efforts that did not include safety precautions have likewise deteriorated in certain factors. The IDHKE is being created in this piece of the proposal, and the data contents are being exchanged securely with the end users from a cloud server, which is being constructed. As a result, the sharing of secret key information is done safely. By choosing one random prime number for each parameter value and master secret key, secure key creation may be accomplished which is difficult for hackers to crack. Secure data transfer is, therefore, guaranteed in the suggested technique of correctness and dependability in the authentication process.

The transparent data encryption feature of Microsoft SQL Server 2008 was introduced with the release of SQL Server 2008. This feature enables the SQL Server to encrypt data as it is written to the server's hard disc, and the SQL Server decrypts data as it is read from the hard drive into the server's memory. The benefit of using this approach is that you are able to encrypt all data without causing any changes to the data in any way. This function also safeguards all of your data when it is backed up since the backup is encrypted as well as the original. Instead of encrypting the data contained inside the blocks of data, this encryption is accomplished by encrypting the blocks of data themselves. It is important to note that when data are encrypted, just the data contained inside the table is encrypted, while TDE will encrypt the metadata about the tables, as well as the white space in the data pages, among other things.

The disadvantage of employing transparent data encryption is that if someone is able to get access to your SQL Server by conventional methods, or by using something such as a SQL Injection, they will still be able to extract the data from your SQL Server by just querying the data in your SQL Server.

Additionally, transparent data encryption will increase the CPU burden on the SQL Server since each data page that is read from or written to the disc must be encrypted. Transparent data encryption is a good thing. On systems with a high level of demand, this might result in a significant increase in CPU resources. Transparent data encryption is a feature that is incredibly simple to enable. Simply right click on the database in SQL Server Management Studio and choose Properties from the drop-down menu. Then, click on the choices tab and go to the bottom of the option list to confirm your selection. When you do this, the transparent data encryption option for this database will be enabled.

### 3.1. Methodology of Secured Data Transmission

**Figure 1:**
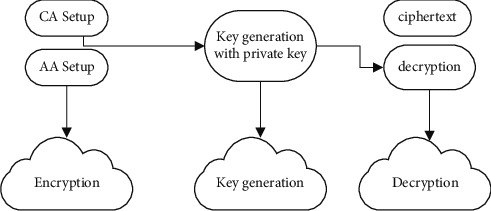
Overall proposed work.

The improved data transmission with secure key algorithm (IDTSK) is developed in this piece of the proposal; the data contents are exchanged securely with the end users from the cloud server, which is being built. As a result, the sharing of secret key information is done safely. By choosing one random prime number for each parameter value and master secret key, secure key creation may be accomplished that is difficult for hackers to crack. As a result, the suggested technique of accuracy and reliability authentication ensures safe data transfer of all information.  Figure 1 represents the overall proposed work.

As outlined below, the suggested approach will be examined in more depth in the following sections.

### 3.2. Overall System Setup

The four main organizations in the system are comprised of four important components: The Central Authority (CA), The Cloud, The Data Owners (DOs), and The Data Receivers (SDRs) [36].

Caution: the cryptographic key is assigned to a user following a set of characteristics, and the Central Authority divides the key into two components or sections (CA). The first is known as a Secret Part Key (SPK), and the second is referred to as a Security Device Key (SDK), and both are used for storing sensitive information in a secure manner inside a limited computer device [35]. Management of each secured user device is the responsibility of CA and the associated SDK. If, for example, during a specific phase of SDK update, a new SDK is generated in the security device's CA, and the encryption key is transmitted to the cloud once again; this is referred to as a “hard reset.”

To guarantee that the data are secured, all encrypted shared data are stored in a semitrusted party in the cloud, with the users' Universal Identity (UID) within the table being saved as well as the encryption key when necessary. In the case that the DR shares encrypted data with the proxy cloud via the DR queries, the proxy cloud re-encrypts the shared data with the appropriate key and sends the encrypted shared data back to the DR. Ownership of data is a term used to describe the sharing of information among data receivers (SDRs) (DO). The CPABE encrypts data before sharing it with other users, in line with the organization's access policy. It is referred to as the user in this case since it is used to refer to the data exchange between a cloud and an individual user. When DR data are shared, decryption from the cloud is required, which results in ciphertext with an entirely new encryption key. SPK decodes SDRs' security device-encrypted ciphertext after determining whether or not SDRs' attribute set access policy has been met. SDK never reveals anything during decryption, and a process of partial decryption is removed out of the security device and performed outside of the security device to ensure that no sensitive information is exposed [32]. Contact with the CA and the use of a new security device if a security device has been lost or stolen are required for the revocation of DR to occur.

In *q* prime order of *H*_l_ bilinear group, *h* is a generator of Hl. Additionally, the bilinear map denotes e: *H*1 × *H*1⟶*H*2. The size of the groups is determined by a security parameter *l*. Define the Lagrange coefficient Δ_*j*,*p*_ for *j* ∈ *A*_*q*_ and a set, *S*, of elements in Aq : Δ_*j*,*p*_(*y*)= organized as CA setup, AA setup, secret key generation, data encryption, and data decryption.

### 3.3. Initial System Setup

The CA setup algorithm needs to run for CA setup as input from a parameter of security. *H* and *H*_*U*_ are a couple of multiplicative groups chosen by CA with similar *p* prime order as well as a bilinear map: *H* × *H*⟶*H*_*U*_, a couple of system parameters as of the *d* maximum tree depth and the maximum node cardinality num.

The following are steps for the algorithm to proceed. Thus, the universe of real attributes *W*={1,…, *m*} and a (num −1)-sized universe of dummy attributes *v*={*m*+1,…, *m*+ num −1}. In sequence, a (*e*, num)−universal access tree *T* is used. Procedures' implicit inputs are assumed to be *e*, num, *v*, *v*^*∗*^, *U*. Now, for each real attribute, *j* ∈ *u*, choose a set of |*U*| numbers {*u*^*∗*^*k*_*x*_}*x* ∈ Φ_*T*_ uniformly at random from *Z*_*p*_. Furthermore, for each dummy attribute *j* ∈ *u*, choose a set of |Φ_*T*_| numbers {*t*_jx_^*∗*^}*x* ∈ Φ_*T*_ uniformly at random from *Z*_*p*_. Finally, choose *y* uniformly at random in *Z*_*p*_. Equations (1) and (2) define the public parameters PK:

(1) $$\begin{array}{c} \text{GPP}=f{\left(h,h\right)}^{z},{\left\{U_{ky}=h^{u_{kz}}\right\}}_{j\in u,x\in \psi_{T}},{\left\{U=h^{u^{\prime }}|y\right\}}_{k\in v,y\in \phi_{u}}^{a}. \end{array}$$

The master key MK is

(2) $$\begin{array}{c} y,{\left\{t_{j,x}\right\}}_{j\in u,x\in \Psi_{T}},{\left\{t_{jx}^{*}\right\}}_{j\in u,x\in \Phi_{T}}. \end{array}$$

CA accepts both user registration and AA registration as forms of registration.

Every user is required to enroll in CA at a system startup to be able to access certain features. A unique identifier (uid) is given to a lawful user on a system by the system administrator. Two random numbers are produced in every user uid, such as *u* “uid Zp with secret keys worldwide and, in turn, produces the global public keys for the following.

AA registration: when the system is first started, the CA must be registered with every AA. On the legal authority system in AA, a global attribute authority is issued worldwide by the CA to identify the AA for the first time. Other global public/secret keys, such as (GPK “uid” “GSK” “uid”), are supplied from CA to each user in conjunction with AA “aid.” In addition, a verification key is provided to AA aul for use in validating the user's certificates issued by the CA once they have been granted. Attributes' set is denoted by *S*_aid_ in every attribute authority of AA_aid_ as 3 equations. Three random numbers *α*_aid_*β*_aid_*γ*_aid_ ∈ Zp are chosen along with the authority secret key:

(3) $$\begin{array}{c} \text{SK aid}=\left(\alpha_{\text{aid}}\beta_{\text{aid}},\gamma_{\text{aid}}\right). \end{array}$$

“aid” represents data encryption, “aid” represents attributes differentiated by “aid” from multiple AAs, and “aid” represents attribute revocation for “aid.” According to (4), the creation of the public key PK “aid” occurs as

(4) $$\begin{array}{c} {\text{PK}}_{\text{aid}}=\left(e{\left(g,g\right)}^{a_{\text{ald}}},g^{\beta_{\text{aid}}},g^{1/\sqrt{I}\text{aid}}\right). \end{array}$$

Equation (5) gives a generation of the public attribute key on every attribute *x*_aid_ ∈ *S*_aid_ as

(5) $$\begin{array}{c} {\text{PK}}_{x_{\text{aid}}}=\left({\text{PK}}_{1x_{\text{aid}}}=H{\left(x_{\text{aid}}\right)}^{v_{x_{\text{aid}}},}{\text{PK}}_{2,x_{\text{aid}}}=H{\left(x_{\text{aid}}\right)}^{v_{x_{\text{ait}}}}\gamma_{\text{aid}}\right), \end{array}$$

where VK_*x*_*aid*__=*v*_*x*_aid__ are the version key of attribute implicitly. All the public attribute keys {PK_*x*_aid_}_}_*x*_aid_∈*s*_aid__ are published on the public bulletin board of the ACA_aid_ incorporated along with public key PK_aid_ of AA_aid_.

### 3.4. Key Generation in the Proposed Structure

Authentication of every user uid is needed on AA_aid_ in before entitling a few attributes. AA_aid_ receives the Certificate (uid) from submission of the user and, in turn, authentication of the user by AA_aid_ via CA offering verification key. On authentication of AA_aid_, attributes' set *S*_uid.aid_ is entitled to the user uid accordingly with its role otherwise administrative domain identity else it exit. In turn, the users' secret key SK_uid,aid_ is generated by AA_aid_ on execution of the algorithm secret key generation SKeyGen. An attribute set of a user uid is considered. A private key *D* is the output of the key generation algorithm enabling *A* to decrypt a message encrypted under a (d, num) bounded access tree *T*′ iff *T*′(*γ*)=1.

The algorithm proceeds as follows. In all users, a polynomial *q*_*x*_ is chosen randomly among every nonleaf node *x* in the universal access tree *T*. From the source of root node *r*, polynomials are selected, *c*_*x*_=mum − 1. The threshold value is maximum than degrees *x* of the polynomial *q*_*x*_ for each *x*. Complete definition of other points of the polynomial *q*_*r*_, randomly, is done as *c*_*r*_ for the root node *r* by fixing qr(0)=*y*.*q*_*x*_ which is defined completely by selecting *c*_*x*_ other points in random for else nonleaf node *x* as fixing *q*_*x*_(0)= *q*_parent_(*x*)(index(*x*)). In a sequence of polynomials determined, secret values to the user are followed as

(6) $$\begin{array}{c} {\left\{D_{jx}=g^{q_{x}\left(j\right)/L_{\text{jx}}}\right\}}_{j\in u,x\in \Psi_{T}},{\left\{D_{j,x}=g^{{4x\left(\theta \right)/r_{jx}}^{\prime }}\right\}}_{j\in u^{*},x\in \Phi_{T}}. \end{array}$$

Decryption key *D* is obtained from the secret values' set. A (*d*, num) bounded access tree *T* are selected first for encryption of a message ∈GPP by the encrypter *ε*. Real attributes are assigned to the leaf nodes in *T*′.

Message *M* with the access tree *T*′ can encrypt. On demand, normal forms get encrypted. A map between the nodes in *T*′ and the universal access tree *T* are defined by *ε*. For each nonleaf node *x* in *T*′, *ε* chooses an arbitrary (num − *k*_*x*_)-sized set *ω*_*x*_ of dummy child nodes of map(*x*) in *T*. Let *f*(*j*, *x*) be a Boolean function such that *f*(0, *x*)=1 if a real attribute *j* ∈ *U* is associated with a leaf child of node *x* ∈ *T* else 0. Choose a random value *s* ∈ *Z*_*p*_, and equation (7) gives the ciphertext *E* as follows:

(7) $$\begin{array}{c} <T^{\prime },E^{\prime }=m>Y^{5},\left\{E_{j,x}=T_{j,\text{map}\left(x\right)}^{s}\right\}j\in u,x\in \Psi_{T^{\prime }}:f\left(j,x\right)=1\left\{E_{j,x}^{*}=T_{j,\text{map}\left(x\right)}^{*s}\right\}j=\text{att}\left(z\right):z\in w_{x},x\in \Phi_{T}(\rangle . \end{array}$$

Any concerned encrypted data are queried from every legal user in the system. Decryption algorithm decrypts ciphertext from the server depending on the data received via secret keys as of various AAs. Ciphertext E defines user satisfied attributes in access structure and so the content key is used by the user. Ciphertext E, D private key, and a node *x* in *T*_0_ give input of a recursive algorithm in decrypt Node (*E*, *D*, *x*). Group element of *G*^2^ or ⊥ are output. Initially, *x* is a leaf node is considered. Let *j*=att(*x*) and *w* be the parent of *x*. Equation (8) represents the decrypt node:

(8) $$\begin{array}{c} \text{if }j\in \gamma \text{ It reduces to }e{\left(g,g\right)}^{\left(s.t{\;}_{j\text{ mape}}w\right)}\text{when }j\in \gamma . \end{array}$$

Now, consider the recursive case when *x* is a nonleaf node in *T*′. The algorithm proceeds as follows. For all nodes *z* that are children of *x*, it calls DecryptNode (E, D, z) and stores the output as *F*_*z*_. Additionally, for each dummy node, *z* ∈ *ω*_*x*_, where *ω*_*x*_ is a select set of dummy nodes of the map (*x*) in *T* chosen by the encrypter, it invokes a function Decrypt Dummy (*E*, *D*, *z*) that are defined below and stores the output as *Fz*. Let *j* be the dummy attribute associated with *z*. Then, (9) shows

(9) $$\begin{array}{c} \text{Decrypt Node}\left(E,D,x\right)=e\left(D^{*}j_{\text{jmap}\left(w\right)},E_{j,x}^{*}\right), \end{array}$$

which reduces to (*g*, *g*)^*s*.4 map(*x*)^(*θ*). Let Ω*x* be an arbitrary *k*_*x*^−^_sized set of child nodes *z* such that *F*_*z*_ ≠ ⊥. Furthermore, let *S*_*x*_ be the union of the sets Ω*x* and *ω*_*x*_. Thus, we have that |*S*_*x*_|= num. Let *g*ˆ=*e*(*g*, *g*). If no *k*_*x*_-sized set *x* exists, then the node *x* was not satisfied and the function returns ⊥. Otherwise, compute the following equations:

(10) $$\begin{array}{c} \prod_{z\in \Omega_{x}}F_{z}^{\Delta_{l}s_{1}^{\prime }\left(a\right)}\prod_{z\in \omega_{x}}F_{z}^{A_{t}\cdot s_{k}^{\prime }\left(0\right)}. \end{array}$$

Return the result. Now that we have defined decrypt node, the decryption algorithm simply invokes it on the root r'of *T*′. Observe that DecryptNode (*E*, *D*, *r*′)=*e*(*g*, *g*)^sy^ iff *T*′(*γ*)=1 and note that

(11) $$\begin{array}{c} F_{r},=e{\left(g,g\right)}^{s.q_{\text{map}\left(r\eta \right)}}\left(0\right)=e{\left(g,g\right)}^{s\cdot q_{r}\left(0\right)}=e{\left(g,g\right)}^{\text{sy}}, \end{array}$$

where *r* is the root of the universal tree T.

Since *E*′=*M* · *e*(*g*, *g*)^sy^, the decryption algorithm simply divides out *e*(*g*, *g*)^sy^ and recovers. *M*.

In asymmetric encryption, in 1976, the key exchange is performed by the Diffie–Hellman algorithm designed by Whitfield Diffie and Martin Hellman. It removes the necessity of transferring keys among a couple of communication parties. A shared secret key is possible to generate every party on data encryption and data decryption. Diffie–Hellman key exchange is done securely as of various security protocols and services in reliable communication. A random parameter is chosen for the algorithm inefficiency. New shared keys are generated randomly for the exchange of information among the receiver as well as the sender and the receiver. Thus, various ciphertexts are generated at a time of a similar message. Several attacks are prevented on utilising this scheme. “q” is a prime number, and “a” is a primitive root which is selected by the basic version of the Diffie–Hellman algorithm on condition q > a. The powers of “a” generate all integers from 1, 2, 3, 4, 5, 6, 7, 8, 9, 10, 11, 12, 13, 14, 15, 16, 17, 18, 19, 20, 21, 22, 23, 24, 25, 26, 27, 28, 29, 30, 31,…. All integers from 1 to p − 1 are generated by aq-1 mod q. In this method, the first user chooses a private key by selecting a random natural number I from a hat. A mod q is used to compute the public key for many users with the same name and password. Similarly, the second user chooses the letter *j* for its private key. It is the same process that was used to create the first user public. The public keys of the two users are then exchanged between them. To calculate the shared secret key, each user utilizes its private key in conjunction with the public keys of the other users. Encryption and decryption are accomplished with the help of this secret key (Algorithm 1).

Because the private keys I and *j* are not sent and thus cannot be intercepted, only the people involved in the communication are aware of them. As a result, only the people involved in the communication may compute the shared secret key. However, there is still no way to authenticate the people who are interacting since their identities are not connected to the keys that they are sharing. As a result, the algorithm is vulnerable to man-in-the-middle assaults. Improved data transmission with secure key algorithm (IDTSK) describes how public key certificates and digital signatures may be used to protect against these types of attacks. Messages can be encrypted multiple times, yielding the exact cryptic text because the shared key remains constant during the session. To discover connections between plaintext and encrypted text, it is necessary to look for patterns that appear often in the ciphertext. To protect against known-plaintext attacks, it is necessary to include a random component in the key agreement procedure so that a new ciphertext is produced even when the same plaintext is encrypted several times.

## 4. Implementation

When compared with the suggested technique, the experimental findings are verified among the existing methodologies. The research performance evaluation is achieved by matching the current technique with a particular parameter on the basis of the data collected. The improved data transmission with secure key algorithm (IDTSK) approach, which was created in an earlier manner, is widely used in public key cryptosystems today. In comparison to the rest of the class, it achieves a superior encryption message identification and public key via ciphertext. The suggested study, IDHKE, is being developed in order to provide substantial security for health-related data. The following criteria are taken into consideration for attaining performance: resource utilization rate, integrity, secrecy, and degree of user satisfaction.

### 4.1. Confidentiality Comparison

Confidentiality values are analyzed among proposed IDHKE against existing methods improved data transmission with secure key algorithm (IDTSK) are tabulated in Table 1. In scenario of delegation ratio 0.9, 98% confidentiality is attained in IDHKE.

**Figure 2:**
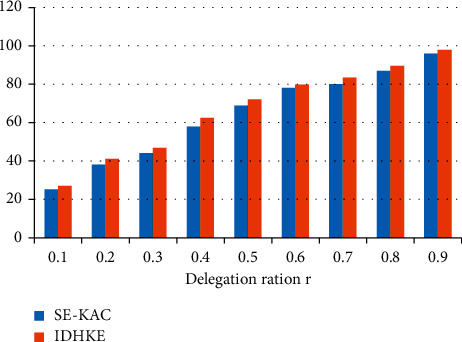
Confidentiality.

The confidentiality results of the prevailing improved data transmission with secure key algorithm (IDTSK) technique against the proposed IDHKE technique are shown in Figure 2. Delegation ratio is represented in *X*-axis and confidentiality is represented by *Y*-axis. The proportion of the delegated cipher text classes to the classes entirely is referred to as the delegation ratio. The IDTSK (improved data transmission with secure key algorithm) methodology, which was created previously, is known for its dual encryption mechanism with an identifier. In the suggested technique IDHKE, secure key sharing is implemented. The practical results prove methodology of IDHKE achieves maximum confidentiality against improved data transmission with secure key algorithm (IDTSK) techniques.

### 4.2. Integrity Comparison

Integrity values are analyzed among proposed IDHKE against existing methods, improved data transmission with secure key algorithm (IDTSK), which are tabulated in Table 2. In the scenario of delegation ratio 0.9, 97.2% integrity is attained in IDHKE.

**Figure 3:**
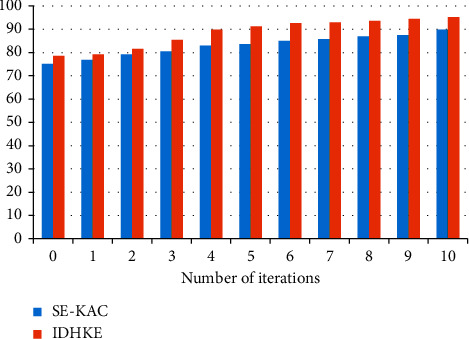
Integrity.

Figure 3 shows the results of the current improved data transmission with the secure key algorithm (IDTSK) method compared to the proposed IDHKE technique in terms of structural integrity. Appendix indicates the pseudocode of the proposed work. The delegation ratio (represented by the *X*-axis) and the confidentiality (represented by the *Y*-axis) are shown on the graph. The delegation ratio refers to the proportion of delegated cipher text classes that are assigned to the classes in their entirety. Whenever it comes to data transmission, one of the most well-known methods is the improved data transmission with secure key algorithm (IDTSK). The suggested method IDHKE includes a secret key management system that is implemented. The practical findings demonstrate that the IDHKE approach delivers maximal integrity when compared to improved data transmission with secure key algorithm (IDTSK) methods.

### 4.3. User Satisfaction Degree

**Figure 4:**
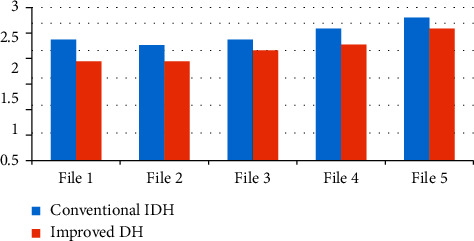
User satisfaction comparison.

User satisfaction levels about cloud resources are referred to as Quality of Service to the user. Subjective and objective services are the two primary services provided to users by resources in which feedback information is required in the approach of subjective beginning users on completion of useful resource, and objective approach offers higher degrees of satisfaction among users' resource demand and the selected resources' capability.  Figure 4 represents the user satisfaction.

Additionally, as shown in Figure 4, this section performs a comparative analysis of both proposed IDHKE and existing improved data transmission with secure key algorithm (IDTSK) approaches for parameter resource consumption rate as well as many other variables. When compared to the existing improved data transmission with secure key algorithm (IDTSK), IDHKE achieves a higher rate of resource utilization than the current improved data transmission with secure key algorithm (IDTSK). With rising resource consumption, the number of users increases from 50 to 300 because user tasks use more resources as a consequence of increased resource utilization. Rather than taking away a higher capacity resource from a user job with a lower priority, this arrangement enables more high capacity resources to be made available to users with higher demands. The upgraded Diffie–Hellman is compared to the traditional Diffie–Hellman in terms of time and memory consumption.

**Figure 5:**
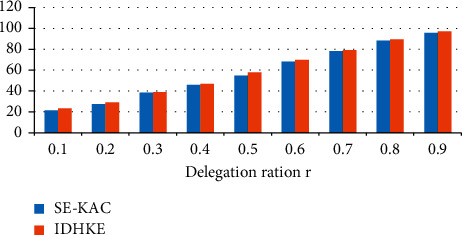
Time comparison between conventional DH and improved DH.

The proposed improved DH beats the existing conventional DH in terms of time consumption while dealing with files of variable length, as shown by the comparison given in Figure 5.

## 5. Conclusion

With the creation of the algorithm of improved Diffie–Hellman key exchange, it is hoped to optimise secret key exchange in a manner that is safe for data receivers while still preserving data security in the future. Because the secret key information must be sent in a secure way in order to preserve the greatest possible level of security. Using a master secret key and a random prime number for each parameter value makes it possible to establish a secure key generation process that is difficult to break. This enables accurate and reliable verification to be carried out during secure data transmission to be completed. Access control limitation is achieved via the use of attribute-based encryption, which is both secure and trustworthy. This platform, known as CloudSim, makes it easier to finish the research method, resulting in better findings than presently accessible techniques [39].

## Figures and Tables

**Algorithm 1:**
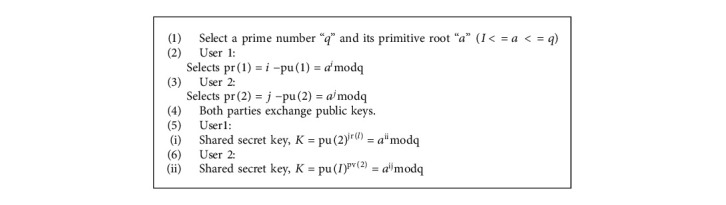
Pseudocode for the proposed algorithm.

**Table 1:** Confidentiality comparison values.

Delegation ratio (%)	IDTSK	IDHKE
0.1	25.2	27
0.2	38	41
0.3	44	46.8
0.4	58	62.5
0.5	69	72
0.6	78	79.8
0.7	80	83.4
0.8	87	89.6
0.9	96	98

**Table 2:** Integrity comparison values.

Integrity (%)		
Delegation ratio (%)	Improved data transmission with secure key algorithm (IDTSK)	IDHKE
0.1	21.4	23.5
0.2	27.5	29
0.3	38.4	39
0.4	45.9	47
0.5	54.9	58
0.6	68.3	69.8
0.7	78.3	79.2
0.8	88.3	89.5
0.9	95.8	97.2

## Data Availability

The data that support the findings of this study are available from the corresponding author upon request.

## Appendix

Key expansion: the key expansion procedure divides a 448-bit key into multiple subkey arrays, resulting in a key with a total size of 4168 bytes when completed. Blowfish makes extensive use of subkeys to accomplish its goals. These keys will be produced in advance of any data encryption or decryption operations taking place.

  The p-array is composed of 18 32-bit subkeys, which are denoted by the letters P1, P2,…, P18.
  S1, 0, S1, 1,…, S1, 255 are four 32-bit S-Boxes with 256 elements each: S1, 0, S1, 1,…, S1.
  S2, 0, S2, 1,…, S2, 255 are all numbers between 0 and 255.
  S3, 0, S3, 1,… S3, 255 S4, 0, S4, 1,… S4, 255 S5, 0, S5, 1,… S5, 255 S6, 0, S6, 1,… S6, 255.
  Divide the number *x* into two 32-bit halves, xL and xR. For I ranging from 1 to 16:
  the product of XL and Pi is xL.
  F(XL) XOR xR = F(XL) XOR xR.
  Swap XL and xR.
  XL and xR should be swapped (reverse the previous exchange), P17 = xR XOR xR XOR P17.
  P18 = xL XOR xL XOR P18.
  Combining xL and xR is recommended.
